# Risk of Bowel Obstruction in Patients Undergoing Neoadjuvant Chemotherapy for High-risk Colon Cancer: A Nested Case-control–matched Analysis of an International, Multicenter, Randomized Controlled Trial (FOxTROT)

**DOI:** 10.1097/SLA.0000000000006145

**Published:** 2023-11-10

**Authors:** FOxTROT Collaborating Group

**Affiliations:** Mr James Glasbey MBBCh, BSc, MRCS, PhD, Institute of Applied Health Research, University of Birmingham, B15 2TH

**Keywords:** case-control study, chemotherapy, colon cancer, colorectal cancer, FOxTROT trial, gastrointestinal cancer, multidisciplinary team, neoadjuvant therapy, obstruction, randomized trial

## Abstract

**Objective:**

This study aimed to identify risk criteria available before the point of treatment initiation that can be used to stratify the risk of obstruction in patients undergoing neoadjuvant chemotherapy (NAC) for high-risk colon cancer.

**Background:**

Global implementation of NAC for colon cancer, informed by the FOxTROT trial, may increase the risk of bowel obstruction.

**Methods:**

A case-control study, nested within an international randomized controlled trial (FOxTROT; ClinicalTrials.gov: NCT00647530). Patients with high-risk operable colon cancer (radiologically staged T3-4 N0-2 M0) that were randomized to NAC and developed large bowel obstruction were identified. First, clinical outcomes were compared between patients receiving NAC in FOxTROT who did and did not develop obstruction. Second, obstructed patients (cases) were age-matched and sex-matched with patients who did not develop obstruction (controls) in a 1:3 ratio using random sampling. Bayesian conditional mixed-effects logistic regression modeling was used to explore clinical, radiologic, and pathologic features associated with obstruction. The absolute risk of obstruction based on the presence or absence of risk criteria was estimated for all patients receiving NAC.

**Results:**

Of 1053 patients randomized in FOxTROT, 699 received NAC, of whom 30 (4.3%) developed obstruction. Patients underwent care in European hospitals including 88 UK, 7 Danish, and 3 Swedish centers. There was more open surgery (65.4% vs 38.0%, *P*=0.01) and a higher pR1 rate in obstructed patients (12.0% vs 3.8%, *P*=0.004), but otherwise comparable postoperative outcomes. In the case-control–matched Bayesian model, 2 independent risk criteria were identified: (1) obstructing disease on endoscopy and/or being unable to pass through the tumor [adjusted odds ratio: 9.09, 95% credible interval: 2.34–39.66] and stricturing disease on radiology or endoscopy (odds ratio: 7.18, 95% CI: 1.84–32.34). Three risk groups were defined according to the presence or absence of these criteria: 63.4% (443/698) of patients were at very low risk (<1%), 30.7% (214/698) at low risk (<10%), and 5.9% (41/698) at high risk (>10%).

**Conclusions:**

Safe selection for NAC for colon cancer can be informed by using 2 features that are available before treatment initiation and identifying a small number of patients with a high risk of preoperative obstruction.

Bowel obstruction is a serious complication of colonic cancer and accounts for 50% of mortality within a year of diagnosis. It is the precipitant for the majority of emergency bowel cancer surgery, which incurs a 3-fold higher risk of death compared with a planned operation.^1–4^ Bowel obstruction also has a detrimental impact on longer-term survival and oncological outcomes.^5,6^

The Fluoropyrimidine, Oxaliplatin and Targeted-Receptor pre-Operative Therapy for patients with high-risk, operable colon cancer (FOxTROT) trial^7^ has demonstrated the safety and efficacy of short-course neoadjuvant chemotherapy (NAC) in patients with high-risk operable colon cancer. A substantial and rapid response to NAC was observed at histopathologic assessment of the resected tumor; up to 60% treated with a 6-week duration of NAC had tumor regression at surgery, which translated into a 25% reduction in recurrent or persistent disease at 2 years, compared with straight to surgery^8^ and NAC can now be considered a therapeutic option in this patient group. However, for patients undergoing NAC, deferring surgery can put patients at risk of colonic obstruction. Oncologists need to be aware of this risk to their patients, as timely management is critical.

As more integrated treatment pathways are developed for high-risk colonic cancer, improved patient stratification for large bowel obstruction risk will be required. Recognizing patient’s risk factors would inform the consent process, enrich multidisciplinary team (MDT) decision-making, and enable targeted active monitoring.

This study aimed to identify clinical, pathologic, radiologic, and endoscopic features of colon cancer that can be used to stratify patients at risk of bowel obstruction. This sought to inform perioperative management of locally advanced colon cancer.

## METHODS

### Study Setting and Design

The FOxTROT trial (ISRCTN 87163246) was an international, multicenter, randomized controlled trial testing the feasibility, safety, and efficacy of preoperative chemotherapy for colon cancer. Patients with radiologically staged locally advanced tumors (cT3 and above) were randomly assigned in a 2:1 ratio to the short course (3 cycles) NAC and standard adjuvant chemotherapy (AC) or standard AC alone.^7,8^ Patients with emergency presentations of colon cancer such as obstruction or perforation were excluded. The inclusion and exclusion criteria of the full trials are available in the published FOxTROT trial protocol. Hospitals managing patients with colon cancer through an MDT in the United Kingdom, Sweden, or Denmark were eligible. This study was a preplanned secondary analysis of FOxTROT data with a nested case-control study. National and institutional approvals were obtained for the FOxTROT trial protocol from the University of Birmingham, the NHS National Research Ethics Service, and all participating international institutions according to relevant local requirements. An Independent Data Monitoring Committee reviewed the database annually.

### Definition of Cases and Controls

Cases were selected according to the following criteria: (1) met inclusion criteria for the FOxTROT^7,8^; (2) randomized to receive NAC and standard postoperative chemotherapy; (3) developed proven or symptomatic colonic obstruction after randomization; (4) diagnosis of obstruction was made before the planned date of surgery. Colonic obstruction was defined pragmatically as (1) proven obstruction, with radiologic and clinical evidence of complete obstruction and/or obstruction requiring radiologic placement of a colonic stent or urgent surgery (within 48 hours of presentation); (2) symptomatic obstruction, where radiologic evidence was inconclusive but with clinical symptoms consistent with obstruction and/or obstruction requiring expedited surgery (>48 hours from presentation).

Controls were defined as patients randomized to the FOxTROT trial to receive NAC and AC but did not develop proven or symptomatic colonic obstruction before their planned date of surgery. Each case was matched with 3 controls (1:3 ratio) based on gender (male or female) and age group (<50, 50–59, 60–69, or ≥70 years). Controls were sampled at random from other (unobstructed) patients receiving NAC using a random number matching algorithm within SAS Software (SAS Institute Inc., Cary, NC, USA).^9^

### Identification of Cases

Cases were identified from serious adverse events reported by site investigators in the FOxTROT trial and corroborated by data from the NAC Case Report Form. Where further detail was required, sites retrieved source data (clinical notes or Electronic Health Records) to confirm or refute the diagnosis of obstruction. The identification of cases from serious adverse event data was performed independently by 2 investigators (J.G., K.H.), and any differences were resolved by the trial Chief Investigator (D.M.).

### Outcome Measures

The primary outcome measure was colonic obstruction, defined as proven or symptomatic obstruction (see Definition of cases), after randomization and before the planned date of surgery. Secondary outcome measures were grouped into 3 categories: (1) surgical decision-making [operative approach (laparoscopic vs open); stoma formation]; (2) pathologic outcomes [resection plane (intramesocolic vs mesocolic vs muscularis propria); other bowel perforation (away from the tumor site); resection margin status (pR0 vs pR1 vs pR2)]; (3) clinical outcomes, defined within 30 days of surgery with a day of surgery as day 0 [death; length of stay (days); reoperation; anastomotic leak (in patients for whom an anastomosis was performed)].

### Covariates and Data Sources

Covariates related to clinical and radiographic features at the time of randomization were extracted from the FOxTROT study database including age, sex, tumor location, and baseline radiologic tumour, node, metastases stage. All radiologists were provided with face-to-face training in the assessment of colonic primary tumors within the FOxTROT trial to standardize reporting. Stricturing disease was defined as annular tumors, with evidence of luminal narrowing (in the absence of upstream dilatation of the colon). Stricturing disease either on radiologic examination or endoluminal evaluation (or both) was coded as “structuring” for the purposes of the risk model. Endoscopic data (annular vs other tumor types, ability to pass endoscope past the tumor site) were not collected routinely in the FOxTROT trial, so were extracted for both cases and controls in source data from collaborating sites. Data extraction was performed from source data by 2 independent investigators (J.G., Y.S.), with any differences resolved by the senior investigator (D.M.). Pathologic data on tumor regression grade (no/mild regression, moderate/marked/complete regression) was extracted from postoperative histologic data, confirmed with a central analysis of all specimens. As biopsy data were not collected routinely within FOxTROT, tumor differentiation and subtype derived from the postoperative pathologic analysis were used as a surrogate for likely biopsy findings.

### Statistical Analysis

The study was conducted according to the STROBE (Strengthening the Reporting of Observational Studies in Epidemiology) extension for case-control studies (Appendix B, Supplemental Digital Content 1, http://links.lww.com/SLA/E942) and reported according to SAMPL (Statistical Analyses and Methods in the Published Literature). Missing data were described and included in summary tables where applicable. Full statistical methodology is reported in Appendix A, Supplemental Digital Content 1, http://links.lww.com/SLA/E942.

Clinical, radiologic, endoscopic, and pathologic characteristics of patients and tumors, and clinical and pathologic outcomes were compared between (analysis 1) cases versus all other (unobstructed) patients randomized to receive NAC; (analysis 2) cases versus matched controls. The timing of obstruction was examined using a continuous variable of time (in days) from randomization to diagnosis of proven or symptomatic obstruction. To explore the effect of treatment response on the timing of obstruction, patients were grouped by their histopathologic assessment of treatment response (no or minimal response vs moderate or marked regression).

### Adjustment for Confounding

We assessed the association between covariates and subsequent bowel obstruction in matched patients using Bayesian hierarchical unconditional (unmatched) logistic regression analysis,^10^ with the diagnosis of bowel obstruction as the primary dependent variable. In this mixed-effects model, both proven and symptomatic obstruction were coded into a single obstruction outcome variable. Models were adjusted using clinically plausible covariables listed above, including the matching variables.^10^ Model coefficients are presented as adjusted odds ratio (OR) and 95% credible intervals (CIs); these can be interpreted similarly to 95% C.I.s but are philosophically distinct.^11^ A sensitivity analysis for the primary model was conducted using proven obstruction only as the dependent variable. Analyses were conducted using R Foundation Statistical Program version 3.1.1 and C-STAN (packages: *finalfit, tidyverse, BRMS*). Model diagnostics were explored using *shinystan*.

### Calculation of Absolute Risk of Obstruction in the Presence of Risk Characteristics

The prevalence of clinical, radiologic, endoscopic, and pathologic characteristics independently associated with risk of obstruction in the Bayesian mixed-effects model (named “risk criteria”) in cases and controls were summarized as percentages. The absolute risk of obstruction in the patients receiving NAC in FOxTROT in the presence or absence of each high-risk feature alone or in combination was estimated for tumors across different locations by assuming consistency in the prevalence of risk criteria in the controls sample and patients randomized to NAC. 95% C.I.s for proportions are provided for all percentage estimates. Cut-offs for very low–risk, low-risk and high-risk groups were defined pragmatically through consensus among the international writing group, based on clinically important thresholds to influence clinical practice.

## RESULTS

**FIGURE 1:**
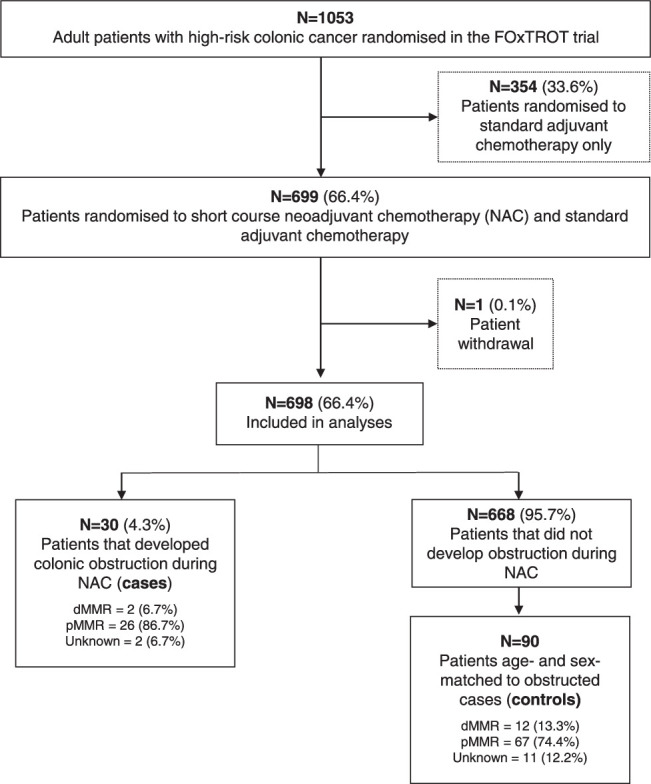
Flowchart of included patients. pMMR indicate mismatch repair proficient.

Of 1053 patients randomized in the FOxTROT trial, 699 (66.4%) were randomized to receive NAC between May 2008 and December 2016. Patients were included from 85 centers (79 in the United Kingdom, 3 in Denmark, and 3 in Sweden). One patient withdrew their data from the study and was subsequently excluded from analyses. Of 698 patients undergoing NAC, 30 (4.3%) developed obstruction of whom 22 (3.2%) had radiologically proven and 8 (1.1%) had progressive symptoms suggestive of obstruction. Figure 1 demonstrates the inclusion of patients in this analysis from the FOxTROT trial participants.

### Natural History of Obstruction

**FIGURE 2:**
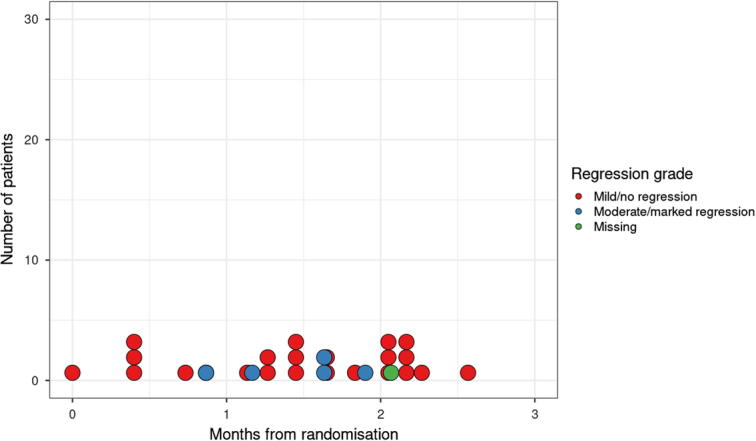
Timing from randomization to obstruction in patients undergoing neoadjuvant chemotherapy grouped by pathologic regression grade.

Figure 2 displays the distribution from the time of randomization to obstruction, grouped by tumor regression grade. The median time from randomization to bowel obstruction was 1.6 months (interquartile range mismatch repair deficient: 1.1–2.0 months). The frequency of obstruction increased over time. There was no clear association between regression grade and timing of obstruction.

One patient was deemed to be obstructed immediately after randomization so was taken straight for surgery and did not start NAC. Of the remaining 29 patients, 20 (69.0%) completed NAC and 9 (31.0%) did not finish NAC. Of those who started NAC, 5 (17.2%) were deemed to have moderate or marked regression, and 23 (79.3%) had mild or no regression (2 missing data). There were 2 patients with mismatch repair deficient tumors among the 30 patients with obstruction (6.7%). We did not identify an association between tumor regression grade (*P*=0.22) or mismatch repair gene status and obstruction (*P*=0.381) in this sample.

#### Analysis 1: Comparison of Obstructed and Unobstructed Patients Receiving NAC

Table 1 displays a comparison of patient and tumor characteristics between the groups. Obstructed patients were more likely to have a tumor at the hepatic flexure (16.7% vs 5.7%), splenic flexure (13.3% vs 2.8%), or in the transverse colon (23.3% vs 7.2%, *P*<0.001) than unobstructed patients. There was a numerically higher proportion of T4 tumors in obstructed patients, but this was not statistically significant (37.9% vs 23.8%, *P*=0.145).

**TABLE 1:** Characteristics of Obstructed Patients (Cases) and Other Unobstructed Patients Randomized to Receive NAC

Factor	Level	Cases (N=30)	Other patients randomized to NAC (N=668)^*^	*P*
Clinical features				
Age at randomization	Mean (SD)	61.6 (9.4)	63.1 (9.9)	0.414
Sex	Female	11 (36.7)	240 (35.9)	1
	Male	19 (63.3)	428 (64.1)	
Tumor location	Cecum	2 (6.7)	119 (17.8)	***<0.001***
	Ascending colon	1 (3.3)	120 (18.0)	
	Hepatic flexure	5 (16.7)	38 (5.7)	
	Transverse colon	7 (23.3)	48 (7.2)	
	Splenic flexure	4 (13.3)	19 (2.8)	
	Descending colon	2 (6.7)	34 (5.1)	
	Sigmoid	7 (23.3)	240 (35.9)	
	Rectosigmoid	2 (6.7)	50 (7.5)	
Baseline radiologic features				
T-stage	Muscularis propria (T2)	0 (0.0	1 (0.2)	0.145
	Beyond muscularis propria (T3)	18 (62.1)	505 (76.1)	
	Adjacent organs or peritoneum (T4)	11 (37.9)	158 (23.8)	
	Missing	1	4	
N-stage	N0	12 (41.4)	157 (23.6)	0.071
	N1 (1–3 nodes)	9 (31.0)	319 (48.0)	
	N2 (4+ nodes)	8 (27.6)	188 (28.3)	
	Missing	1	4	
Maximum tumor thickness (mm)	Mean (SD)	23.9 (21.7)	20.3 (11.7)	0.129
Maximum distance of spread beyond muscularis propria (mm)	Mean (SD)	9.9 (9.0)	9.2 (7.9)	0.622
Irregularly enhancing LNs	Mean (SD)	1.2 (1.8)	1.9 (2.1)	0.123
Peritonealization	Nonperitonealized	3 (13.0)	163 (30.0)	0.129
	Peritonealized	20 (87.0)	380 (70.0)	
	Missing	7	125	
Extramural vascular invasion	No	10 (34.5)	270 (41.0)	0.322
	Minimal spreading	10 (34.5)	190 (28.8)	
	Nodular spread into small vessel	9 (31.0)	151 (22.9)	
	Spread along large vein	0 (0.0)	48 (7.3)	
	Missing	1	9	
Pathologic features				
RAS status	Mutant	6 (27.3)	165 (34.0)	0.730
	Not determined	1 (4.5)	13 (2.7)	
	Wildtype	15 (68.2)	307 (63.3)	
	Missing	8	183	
Tumor subtype	Adenocarcinoma	27 (96.4)	550 (87.5)	0.209
	Mucinous	1 (3.6)	82 (11.4)	
	Signet ring	0 (0.0)	8 (1.1)	
	Missing	2	28	
Differentiation	Well/moderate	27 (96.4)	532 (85.3)	0.168
	Poor	1 (3.6)	92 (14.7)	
	Missing	2	44	

χ^2^ test calculations exclude missing data.

* One patient randomized to NAC and AC withdrew from the FOxTROT trial and was excluded.

Bold and italics values indicate *P*<0.05.

LN indicates lymph nodes; RAS, rat sarcoma gene.

### Outcomes of Obstruction

Of the obstructed patients (n=30), no perforation with frank peritonitis was seen at the operation. Microperforation (contained and sealed) was seen in 5 patients (16.7%); representing a low absolute risk in patients undergoing NAC [1 in 139 (5/698)]. Obstruction was managed with colonic stenting for 8 patients (26.7%) and expedited surgery for 21 (70.0%). One patient died preoperatively of an occlusive stroke; site investigators reported a concurrent symptomatic obstruction in this patient. All other patients (n=29) went on to primary tumor resection.

Table 2 displays the outcomes of surgery in obstructed versus unobstructed patients. There was an increased frequency of open surgery (65.4% vs 38.0%, *P*=0.01) and occurrence of pR1 resections (12.0% vs 3.8%, *P*=0.004) in the obstructed group; however, only one pR2 resection was observed in an obstructed patient. There were no significant differences observed in the rates of stoma formation, anastomotic leak, reoperation, overall recurrence, or death up to 30 days after surgery.

**TABLE 2:** Clinical and Pathologic Outcomes in Obstructed Patients (Cases) Versus Other Unobstructed Patients Randomized to Receive NAC

Outcome	Levels	Cases (N=30)^*^	Other patients randomized to NAC (N=668)	*P*
Surgical decision-making				
Operative approach	Open	17 (65.4)	216 (38.0)	***0.01***
	Laparoscopic	9 (34.6)	352 (62.0)	
	Missing	4	100	
Stoma formation	No	24 (88.9)	569 (88.4)	1
	Yes	3 (11.1)	75 (11.6)	
	Loop stoma	2 (66.7)	45 (60.0)	1
	End stoma	1 (33.3)	30 (40.0)	
	Missing	3	24	
Pathologic outcomes				
Resection plane	Mesocolic	16 (76.2)	133 (86.2)	0.324
	Intramesocolic	4 (19.0)	62 (11.0)	
	Muscularis propria	1 (4.8)	16 (2.8)	
	Missing	9	105	
Bowel perforation^†^	No	26 (86.7)	615 (92.2)	0.454
	Yes	4 (13.3)	52 (7.8)	
	Missing	3	70	
Margin status	pR0	21 (84.0)	577 (95.8)	***0.004***
	pR1	3 (12.0)	23 (3.8)	
	pR2	1 (4.0)	2 (0.3)	
	Missing	5	66	
Clinical outcomes (up to 30 postoperative days)				
Death	No	27 (96.4)	651 (99.5)	0.396
	Yes	1 (3.6)	3 (0.5)	
	Missing	2	14	
Length of stay	Mean (SD)	10.8 (15.1)	7.3 (7.5)	***0.023***
Reoperation	No	27 (96.4)	626 (95.7)	1
	Yes	1 (3.6)	28 (4.3)	
	Missing	2	14	
Anastomotic leak	No	27 (96.4)	617 (96.7)	1
	Yes	1 (3.6)	21 (3.3)	
	No anastomosis^‡^	1	30	
	Missing	2	14	
Adjuvant therapy				
Treatment status	Completed	10 (33.3)	436 (65.3)	***<0.001***
	Started did not finish	10 (33.3)	135 (20.2)	
	Did not start	10 (33.3)	80 (12.0)	
	Missing	0	17	
Oncologic outcomes (at 2 y after randomization)				
Overall recurrence	No	22 (75.9)	549 (82.2)	0.599
	Yes	7 (24.1)	119 (17.8)	

* One case died preoperatively of an occlusive stroke, so postoperative outcome data are not available.

† Included both macroscopic perforation (noted at operation) and microscopic (noted during pathologic examination).

‡ Patients with no anastomosis not included in proportion of patients with anastomotic leak. χ^2^ test calculations exclude missing data.

Bold and italics values indicate *P*<0.05.

Obstructed patients were less likely to start AC than nonobstructed patients (70.0% vs 88.0%, *P*<0.001), and fewer who successfully completed 18 weeks of AC (30.0% vs 65.3%, *P*=0.015). The 2-year overall recurrence rate was numerically higher in obstructed versus unobstructed patients, but this was not statistically significant [23.3% (7/30) vs 17.8% (119/668); *P*=0.599].

#### Analysis 2: Comparison of Obstructed Cases and Unobstructed Controls

Table 3 describes the clinical, radiologic, and endoscopic features of the cases and controls. Cases and controls were well matched on both age and sex. Cases were more likely to be observed to have:

1. obstructing disease on baseline endoscopy and/or being unable to pass past the lumen with the endoscope (53.3% vs 20.0%, *P*=0.008);
2. stricturing disease on baseline radiology or endoscopy (78.3% vs 26.2%, *P*=0.002).

**TABLE 3:** Clinical, Radiologic, Endoscopic, and Pathologic Features of Obstructed Cases Versus Matched Controls

Factor	Levels	Cases (N=30)	Controls (N=90)	*P*
Clinical features				
Age at randomization	Mean (SD)	61.6 (9.4)	61.6 (9.6)	0.974
Sex	Female	11 (36.7)	33 (36.7)	1
	Male	19 (63.3)	57 (63.3)	
Tumor location	Caecum	2 (6.7)	13 (14.4)	0.051
	Ascending colon	1 (3.3)	12 (13.3)	
	Hepatic flexure	5 (16.7)	8 (8.9)	
	Transverse colon	7 (23.3)	11 (12.2)	
	Splenic flexure	4 (13.3)	2 (2.2)	
	Descending colon	2 (6.7)	3 (3.3)	
	Sigmoid	7 (23.3)	35 (38.9)	
	Rectosigmoid	2 (6.7)	6 (6.7)	
Baseline radiologic features				
T-stage	Beyond muscularis propria (T3)	18 (62.1)	71 (78.9)	0.117
	Adjacent organs or peritoneum (T4)	11 (37.9)	19 (21.1)	
	Missing	1	0	
N-stage	N0	12 (41.4)	21 (23.3)	0.07
	N1 (1–3 nodes)	9 (31.0)	49 (54.4)	
	N2 (4+ nodes)	8 (27.6)	20 (22.2)	
	Missing	1	0	
Maximum tumor thickness (mm)	Mean (SD)	23.9 (21.7)	20.2 (12.0)	0.257
	Missing	1	1	
Maximum distance of spread beyond muscularis propria (mm)	Mean (SD)	9.9 (9.0)	8.1 (7.2)	0.248
	Missing	1	1	
Irregularly enhancing LNs	Mean (SD)	1.2 (1.8)	1.9 (2.1)	0.151
	Missing	2	2	
Peritonization	Nonperitonealized	3 (13.0)	20 (27.0)	0.273
	Peritonealized	20 (87.0)	54 (73.0)	
	Missing	1	0	
Extramural vascular invasion	No	10 (34.5)	37 (41.1)	0.26
	Minimal spreading	10 (34.5)	29 (32.2)	
	Nodular spread into small vessel	9 (31.0)	17 (18.9)	
	Spread along large vein	0 (0.0)	7 (7.8)	
	Missing	1	0	
Circumferential (radiology)	No	18 (78.3)	54 (70.1)	0.619
	Yes	5 (21.7)	23 (29.9)	
	Missing	7	13	
Stricturing (radiology)	No	13 (56.5)	68 (88.3)	***0.002***
	Yes	10 (43.5)	9 (11.7)	
	Missing	7	13	
Obstructing (radiology)	No	20 (87.0)	76 (98.7)	0.055
	Yes	3 (13.0)	1 (1.3)	
	Missing	7	13	
Pathologic features				
MMR status	Proficient	26 (96.3)	67 (84.8)	0.116
	Deficient	1 (3.7)	12 (15.2)	
	Missing	3	11	
RAS status	Mutant	6 (27.3)	19 (26.8)	0.996
	Not determined	1 (4.5)	3 (4.2)	
	Wildtype	15 (68.2)	49 (69.0)	
	Missing	8	19	
Tumor subtype	Adenocarcinoma	27 (96.4)	70 (84.3)	0.219
	Mucinous	1 (3.6)	12 (14.4)	
	Signet ring	0 (0.0)	1 (1.2)	
	Missing	2	7	
Differentiation	Well/moderate	27 (96.4)	72 (86.7)	0.282
	Poor	1 (3.6)	11 (13.3)	
Endoscopic features				
Unable to pass scope	No	3 (10.0)	25 (27.8)	***0.004***
	NA—Cecal	3 (10.0)	17 (18.9)	
	NA—Flexible sigmoidoscopy only	8 (26.7)	30 (33.3)	
	Yes	16 (53.3)	18 (20.0)	
Circumferential (endoscopy)	No	13 (59.1)	43 (71.7)	0.414
	Yes	9 (40.9)	17 (28.3)	
	Not available	9	30	
Stricturing (endoscopy)	No	6 (27.3)	41 (69.5)	***0.002***
	Yes	16 (72.7)	18 (30.5)	
	Not available	8	31	
Ulcerating (endoscopy)	No	18 (81.8)	47 (78.3)	0.97
	Yes	4 (18.2)	13 (21.7)	
	Not available	8	30	
Polypoid (endoscopy)	No	20 (90.9)	48 (78.7)	0.34
	Yes	2 (9.1)	13 (21.3)	
	Not available	1	29	
Obstructing (endoscopy)	No	14 (60.9)	71 (88.8)	***0.005***
	Yes	9 (39.1)	9 (11.2)	
	Not available	7	10	
Summary baseline features (radiologic and endoscopic)				
Circumferential (all)	No	12 (48.0)	44 (53.7)	0.789
	Yes	13 (52.0)	38 (46.3)	
	Missing	5	4	
Stricturing (all)	No	5 (21.7)	59 (73.8)	***<0.001***
	Yes	18 (78.3)	21 (26.2)	
	Missing	7	10	

χ^2^ test calculations exclude missing or unavailable data.

Bold and italics values indicate *P*<0.05.

LN indicates Lymph nodes; MMR, Mismatch repair gene; RAS, Rat sarcoma gene.

There were numerically more cases at the flexures and in the transverse colon than controls, although this was not statistically significant (*P*=0.051). This relationship could be explained through more frequent occurrence of endoscopic obstruction (*P*=0.004) or stricturing disease in these locations (*P*=0.006, Supplementary Figure 1, Supplemental Digital Content 1, http://links.lww.com/SLA/E942). There was also a trend toward an increased rate of radiologic T4 disease in cases (*P*=0.056).

In the Bayesian mixed-effects model (Table 4, Supplemental Digital Content Table 1, http://links.lww.com/SLA/E942, Supplemental Digital Content Figure 2, http://links.lww.com/SLA/E942, and Supplemental Digital Content Figure 3, http://links.lww.com/SLA/E942), the 2 features that remained strongly associated with obstruction after risk adjustment were:

1. obstructing disease on endoscopy and/or being unable to pass through the lumen with the endoscopy (OR: 9.09, 95% CI: 2.34–39.66);
2. stricturing disease on radiology or endoscopy (OR: 7.18, 95% CI: 1.84–32.34).

**TABLE 4:** Bayesian Unconditional Mixed-effects Model Demonstrating Features Associated With Obstruction in the Case-control–matched Data

		95% credible interval	
	Odds ratio	Lower	Upper
Age, y			
	0.97	0.91	1.05
Sex			
Female	—	—	—
Male	1.63	0.39	7.29
Tumor location^*^			
Ascending or descending colon	—	—	—
Hepatic or splenic flexure	1.39	0.18	9.83
Transverse colon	0.55	0.05	4.99
Sigmoid or rectosigmoid	0.36	0.06	2.25
Radiological T-stage			
T3	—	—	—
T4	1.61	0.39	6.60
Stricturing disease			
No	—	—	—
Yes	***7.18***	***1.84***	***32.34***
Obstructing (endoscopy) or unable to pass scope			
No	—	—	—
Yes	***9.09***	***2.34***	***39.66***

* Tumor locations were grouped anatomically by peritoneal covering of associated the large bowel: sigmoid and transverse on a mesentery; ascending/descending colon retroperitoneal; flexures tethered.

Bold and italics values indicate *P*<0.05.

There was no independent association between tumor location or T-stage and obstruction. This was consistent across sensitivity analyses, including for proven obstruction only (Supplemental Digital Content Table 2, http://links.lww.com/SLA/E942). These were defined as “risk criteria” for the remainder of this analysis.

### Risk Criteria in Cases and Controls

The prevalence of both risk criteria in cases and controls can be found in Supplemental Digital Content Table 3, http://links.lww.com/SLA/E942. A high proportion of obstructed cases had one or more risk criteria (28/30), and half had both risk criteria (15/30). Over half the controls had neither (48/90). Of patients with available data for both parameters (N=77), 14 patients were reported to have stricturing disease and 40 not to have stricturing disease on both radiologic and endoscopic evaluation. Four patients were reported to have stricturing disease on radiologic and not on endoscopic evaluation, and 19 patients on endoscopic but not on radiologic evaluation. There was 59.7% agreement between modalities with a Cohen κ value of 0.37.

### Risk Stratification and Implementation

The estimated proportion of patients randomized to NAC with one or both risk criteria and the rate of obstruction is summarized by tumor location in Table 5. The baseline risk for patients with neither high-risk feature was 0.2% (0.0%–0.6%) across different tumor locations. Identification of one high-risk feature increased the obstruction risk (0.0%–9.9%). The presence of both risk criteria concurrently conveyed the highest risk of obstruction. There was considerable variation in the absolute obstruction risk by tumor location: tumors at the flexures carried the highest risk (67.8%, 95% C.I.: 34.3%–93.8%) while sigmoid or rectosigmoid tumors had the lowest risk (7.6%, 95% C.I.: 0.0%–15.5%). Three risk classifications were defined for all patients undergoing NAC according to the presence or absence of these criteria: 63.4% (443/698) of patients were at very low risk (<1%), 30.7% (214/698) at low risk (1%–10%), and 5.9% (41/698) at high risk (>10%).

**TABLE 5:**
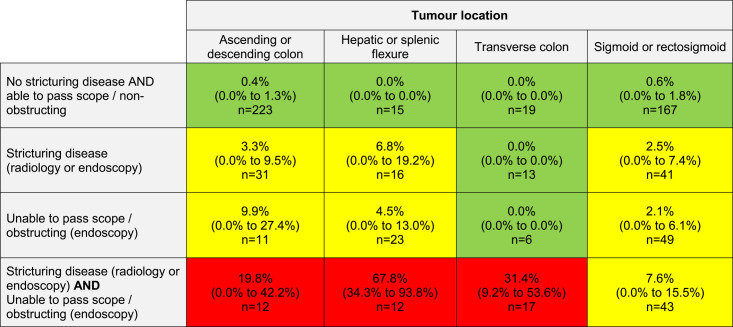
Estimated Absolute Risk of Obstruction in All Patients Receiving NAC in FOxTROT (n=698) With the Presence or Absence of Risk Criteria

Presented as estimated percentage risk with 95% C.I.s in rounded brackets. N represents the estimated number of patients from all patients receiving NAC in FOxTROT represented by each group. A suggested classification is presented by color: green [very low risk <1%, including 443 patients (63.4%)]; yellow [low risk 1% to 10%, including 214 patients (30.7%)]; red [high risk >10%, including 41 patients (5.9%)].

## DISCUSSION

This nested case-control study within an international randomized trial identifies 2 risk criteria features that target a small group of patients (5.9%) at substantial risk of colonic obstruction during NAC for colon cancer. There was considerable variation in the absolute risk of obstruction by tumor site. Importantly, these features are readily available to the MDT before treatment initiation and can be used to inform NAC decision-making. We propose that these data can be used in 4 ways. First, to inform patient consent. Second, to provide enhanced monitoring for patients at risk. Third, to inform a decision to proceed straight to surgery, if appropriate. Fourth, to provide support for colonic stenting or diversion to facilitate NAC, particularly where there is concern that the primary tumor may be unresectable.

Uniquely, this study was able to prospectively observe a large patient cohort who had a planned delay before undergoing resectional surgery. We did not detect an association between the occurrence and timing of obstruction and treatment response (assessed using tumor regression grade) or mismatch repair deficient status. The study did however identify physical tumor factors identifiable by endoscopy and radiology that could define tumors at higher risk of obstruction. As care pathways for colon cancer increase in complexity, this study has implications for the safe implementation of novel chemotherapy pathways for colon cancer.^8,12^

The 2 risk criteria identified here are anatomic properties of a colonic tumor, rather than related to their treatment response, histopathologic subtype, or genomic profile. Specifically, transmural disease (causing stricturing and scarring) is noted on radiology or endoscopy, and an obstructing phenotype is noted at the point of endoscopy. Obstruction was most common in tumors at the hepatic and splenic flexures; it is plausible that this is related to peritoneal tethering and reduced compliance of the colon in these locations, although multivariable analysis suggested tumor stricturing and/or obstructing disease were the most influential features. A description of obstructive features at endoscopy before patients undergo NAC has not previously been reported. We suggest that a complete luminal assessment could be added to MDT assessment criteria for high-risk colon cancer. Being unable to traverse a tumor should not be considered a contraindication to NAC in the absence of clinical symptoms suggestive of acute obstruction [only 3 of 28 patients (10.7%) where this was attempted went on to obstruct]. With the high rate of tumor regression seen in this higher-risk group may in fact benefit most from NAC where the tumor is chemo-sensitive. The presence of a circumferential tumor alone was not associated with the risk of obstruction during NAC, but where it had reached the point that a circumferential tumor caused luminal stricturing that was visible radiologically or endoscopically, this reached statistical significance. As would be expected in a pragmatic study, with a degree of subjectivity in tumor evaluation despite quality assurance measures, there was some disagreement in characteristics reported using different treatment modalities. This highlights the importance of having all information related to multimodal assessment available to the MDT at the time a treatment decision is made.

The rate of stoma formation, anastomotic leak, reoperation, and early postoperative mortality were all comparable between obstructed and nonobstructed patients. This contrasts with a wealth of previous literature.^1,3,5,13,14^ We hypothesize that the favorable outcomes we have seen may be related to the enhanced monitoring provided to patients attending the hospital for neoadjuvant therapy, enabling early intervention in the event of obstructive signs and symptoms. Importantly, this benefit should continue to be realized in routine practice beyond the trial itself.^15^ Improved perioperative outcomes (eg, reduced rates of an anastomotic leak) were also seen following NAC in the FOxTROT trial in comparison with patients randomized to proceed directly to surgery.^8^ This may also reflect the benefits of preoperative patient care in the oncology outpatient setting.^7,8^

There were still adverse outcomes from obstruction observed in this series. The rate of initiation or completion of adjuvant therapy was lower in obstructed patients, which may reflect prolonged recovery after urgent surgery. Although there was no difference in advanced lymph node involvement (N2 rate in analysis 1: 27.6% obstructed cases vs 28.3% unobstructed patients randomized to NAC), there was a higher proportion of T4 rather than T3 tumors seen in obstructed patients (analysis 1: 37.9% vs 23.8% respectively). This may, in part, be due to poorly responsive disease, but potentially may represent an increased propensity for obstruction in more advanced disease. A higher proportion of T4 tumors may also explain the increased pR1 rate in obstructed patients. For patients developing progressive obstruction where there is a real concern for the resectability of the primary tumor, colonic defunctioning or stenting may be helpful to facilitate NAC.^16,17^

This nested case-control study benefitted from high-quality data monitoring, governance, and quality assurance within a randomized trial, and provides the best available evidence on this topic. Nonetheless, this study has several limitations. First, the absolute number of obstructions within this cohort was low (n=30), so inferential statistics are challenging. To account for this, we have adopted the Bayesian methodology to allow us to interpret the probabilistic distributions of factors associated with obstruction. All model assumptions were met, Markov chain Monte Carlo chains demonstrated no evidence of divergence, and the results were robust to sensitivity analyses. Second, case-control matching was performed using only 2 simple matching variables (age and sex) in a 3:1 ratio. This was done to ensure that no factors highly associated with obstruction were included in the case-matching process, therefore becoming uninterpretable. However, this pragmatic approach may have left residual sampling bias or confounding. There are several biases of conditional logistic regression that come under criticism, so unmatched logistic regression was selected for the primary analysis.^10^ Third, the true impact of treatment response on the risk of obstruction may be left unexplored here, as tumors that were highly anatomically unfavorable (ie, obstructed early within the window to surgery) would not have had the opportunity to demonstrate regression at the time of resection; for example, no tumors demonstrating a pathologic complete response obstructed. However, tumors obstructed throughout the treatment window even when displaying moderate regression, suggesting that this did not seem to be a key factor in its pathoetiology. Fourth, the estimates of absolute risk rely on the assumption that the prevalence of risk criteria is similar in the control sample to the other unobstructed patients who received NAC. Fifth, while clinical and radiologic data were collected prospectively, endoscopic characteristics were collected retrospectively [directly from prospectively recorded source data (e.g., endoscopy reports)]. Finally, we were unable to compare outcomes for patients who were not randomized in the trial because of obstructive symptoms with those in the trial who developed obstruction. This was because of a lack of consent and such patients would frequently be managed through an emergency pathway; the generalizability of our data relies on the assumption of similar disease biology.

This study defines a prospectively identifiable subgroup of patients at >10% risk of obstruction and so provides a risk stratification tool that can assist oncologists in the safer introduction of NAC for patients with colon cancer.

## Supplementary Material

## References

[1] Dahdaleh FS Sherman SK Poli EC . Obstruction predicts worse long-term outcomes in stage III colon cancer: a secondary analysis of the N0147 trial. Surgery. 2018;164:1223–1229. 30297240 10.1016/j.surg.2018.06.044

[2] Koebrugge B Vogelaar FJ Lips DJ . The number of high-risk factors is related to outcome in stage II colonic cancer patients. Eur J Surg Oncol. 2011;37:964–970. 21930361 10.1016/j.ejso.2011.08.135

[3] Manceau G Mege D Bridoux V . Emergency surgery for obstructive colon cancer in elderly patients: results of a multicentric cohort of the French National Surgical Association. Dis Colon Rectum. 2019;62:941–951. 31283592 10.1097/DCR.0000000000001421

[4] Webster PJ Tavangar Ranjbar N Turner J . Outcomes following emergency colorectal cancer presentation in the elderly. Colorectal Dis. 2020;22:1924–1932. 32609919 10.1111/codi.15229

[5] Biondo S Gálvez A Ramírez E . Emergency surgery for obstructing and perforated colon cancer: patterns of recurrence and prognostic factors. Tech Coloproctol. 2019;23:1141–1161. 31728784 10.1007/s10151-019-02110-x

[6] Fahim M Dijksman LM van der Nat P . Increased long-term mortality after emergency colon resections. Colorectal Dis. 2020;22:1941–1948. 32627889 10.1111/codi.15238

[7] Dion Morton Matthew Seymour Laura Magill . FOxTROT Collaborative Group . Preoperative Chemotherapy for Operable Colon Cancer: Mature Results of an International Randomized Controlled Trial. J Clin Oncol. 2023;41:1541–1552 36657089

[8] Morton D Seymour MT , FOxTROT-Collaborative-Group . FOxTROT: an international randomised controlled trial in 1052 patients (pts) evaluating neoadjuvant chemotherapy (NAC) for colon cancer. Presented at American Society of Clinical Oncology (ASCO), Chicago, Illinois. 2019. Accessed September 22, 2020. https://ascopubs.org/doi/abs/10.1200/JCO.2019.37.15_suppl.3504 10.1016/S1470-2045(12)70348-0 PMC3488188 23017669

[9] Mortensen LQ Andresen K Burcharth J . Matching cases and controls using SAS® Software. Code Front Big Data. 2019;2:4. 33693327 10.3389/fdata.2019.00004 PMC7931898

[10] Pearce N . Analysis of matched case-control studies. Bmj. 2016;352:i969. 26916049 10.1136/bmj.i969 PMC4770817

[11] Makowski D Ben-Shachar M Lüdecke D . bayestestR: describing effects and their uncertainty, existence and significance within the Bayesian framework. J Open Source Softw. 2019;4:1541.

[12] Karoui M Gallois C Piessen G . Does neoadjuvant FOLFOX chemotherapy improve the prognosis of high-risk Stage II and III colon cancers? Three years’ follow-up results of the PRODIGE 22 phase II randomized multicentre trial. Colorectal Dis. 2021;23:1357–1369. 33580623 10.1111/codi.15585

[13] Mege D Manceau G Beyer-Berjot L . Surgical management of obstructive right-sided colon cancer at a national level results of a multicenter study of the French Surgical Association in 776 patients. Eur J Surg Oncol. 2018;44:1522–1531. 30041941 10.1016/j.ejso.2018.06.027

[14] Winner M Mooney SJ Hershman DL . Management and outcomes of bowel obstruction in patients with stage IV colon cancer: a population-based cohort study. Dis Colon Rectum. 2013;56:834–843. 23739189 10.1097/DCR.0b013e318294ed6b PMC4507563

[15] Downing A Morris EJ Corrigan N . High hospital research participation and improved colorectal cancer survival outcomes: a population-based study. Gut. 2017;66:89–96. 27797935 10.1136/gutjnl-2015-311308 PMC5256392

[16] Harvey PR Rees J Baldwin S . Outcomes of colorectal stents when used as a bridge to curative resection in obstruction secondary to colorectal cancer. Int J Colorectal Dis. 2019;34:1295–1302. 31175420 10.1007/s00384-019-03302-5

[17] Hill J Kay C Morton D . CREST: randomised phase III study of stenting as a bridge to surgery in obstructing colorectal cancer—results of the UK ColoRectal Endoscopic Stenting Trial (CREST). J Clin Oncol. 2016;34(15_suppl):3507–3507.

