# Supplementary material for: Risk of Bowel Obstruction in Patients Undergoing Neoadjuvant Chemotherapy for High-risk Colon Cancer: A Nested Case-control–matched Analysis of an International, Multicenter, Randomized Controlled Trial (FOxTROT)
Source: Ann Surg. 2023 Nov 10;280(2):283–93. doi: 10.1097/SLA.0000000000006145 (PMC11224564; doi:10.1097/SLA.0000000000006145)
Supplement: Supplementary file 1 [file sla-280-283-s001.docx]

**Supplementary table 1.** Posterior summary statistics for primary model of factors associated with postoperative pulmonary complications (summarised in *Table 4*.

|  | | | | | | | **Quantile estimates** | | | | |
| --- | --- | --- | --- | --- | --- | --- | --- | --- | --- | --- | --- |
| Factor | Level | n_eff | Rhat | mean | mcse | sd | 2.50% | 25% | 50% | 75% | 97.50% |
| Age |  | 3,000 | 1 | 0 | 0 | 0 | -0.1 | -0.1 | 0 | 0 | 0 |
| Sex | Male | 3,000 | 1 | 0.5 | 0 | 0.8 | -0.9 | 0 | 0.5 | 1 | 2 |
| Tumour site | Flexures | 1,759 | 1 | 0.3 | 0 | 1 | -1.7 | -0.3 | 0.4 | 1 | 2.3 |
|  | Transverse colon | 1,858 | 1 | -0.6 | 0 | 1.2 | -3.1 | -1.3 | -0.6 | 0.1 | 1.6 |
|  | Rectosigmoid | 1,770 | 1 | -1 | 0 | 0.9 | -2.9 | -1.6 | -1 | -0.4 | 0.8 |
| Radiological T-stage | T4 | 3,000 | 1 | 0.5 | 0 | 0.7 | -0.9 | 0 | 0.5 | 1 | 1.9 |
| Stricturing (all) | Stricturing | 2,195 | 1 | 2 | 0 | 0.7 | 0.6 | 1.5 | 1.9 | 2.5 | 3.5 |
| Obstructing (endoscopy) | Obstructing | 2,517 | 1 | 2.2 | 0 | 0.7 | 0.9 | 1.7 | 2.2 | 2.7 | 3.7 |

n_eff = effective sample size, Rhat = R statistic, mean = point estimate, mcse = standard error of the mean**.**

**Supplementary table 2.** Sensitivity analysis for main model, including only patients with proven colonic obstruction in the definition of the primary outcome.

|  | |  | **95% credible interval** | |
| --- | --- | --- | --- | --- |
|  |  | **Odds ratio** | *Lower* | *Upper* |
| Age | Years | 0.93 | 0.85 | 1.02 |
| Sex | Female | - | - | - |
|  | Male | 2.67 | 0.50 | 18.10 |
| Tumour location | Right or left colon | - | - | - |
|  | Flexures | 1.23 | 0.11 | 15.31 |
|  | Transverse colon | 0.23 | 0.01 | 3.92 |
|  | Sigmoid | 0.64 | 0.07 | 5.94 |
| Radiological T-stage | T3 | - | - | - |
|  | T4 | 1.71 | 0.22 | 8.53 |
| Stricturing disease | No | - | - | - |
|  | Yes | ***6.83*** | ***1.21*** | ***40.4*** |
| Obstructing (endoscopy)  or unable to pass scope | No | - | - | - |
|  | Yes | ***24.55*** | ***4.00*** | ***199.42*** |

Model diagnostics for Rhat, posterior summary statistics and trace plots remained satisfactory in this sensitivity analysis, demonstrating convergence.

**Supplementary table 3.** Proportion of patients with risk criteria in obstructed cases in comparison to unobstructed controls.

|  | **Proportion of patients with risk criteria** | |
| --- | --- | --- |
| **Risk feature** | *Proportion of obstructed cases* | *Proportion of unobstructed controls* |
| No stricturing disease AND  able to pass scope / non-obstructing | 6.7%  (2/30) | 53.3%  (48/90) |
| Stricturing disease* | 78.3%  (18/23) | 26.2%  (21/80) |
| Obstructing (endoscopy) or unable to pass scope | 60.0%  (18/30) | 23.3%  (21/90) |
| Stricturing disease AND  unable to pass scope / obstructing | 50.0%  (15/30) | 11.1%  (10/90) |

*Radiological reports missing for 7 patients

**Supplementary Figure 1.** Interaction between tumour location and proportion of patients with high-risk features: (1) stricturing disease on radiology or endoscopy; (2) unable to pass scope through tumour on endoscopy.

**
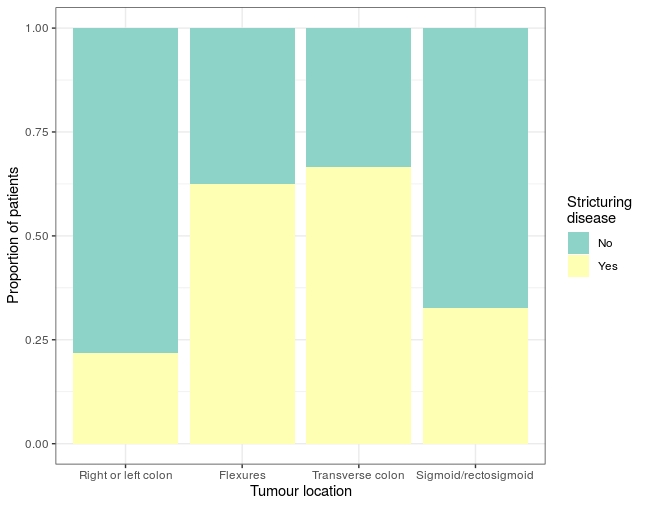

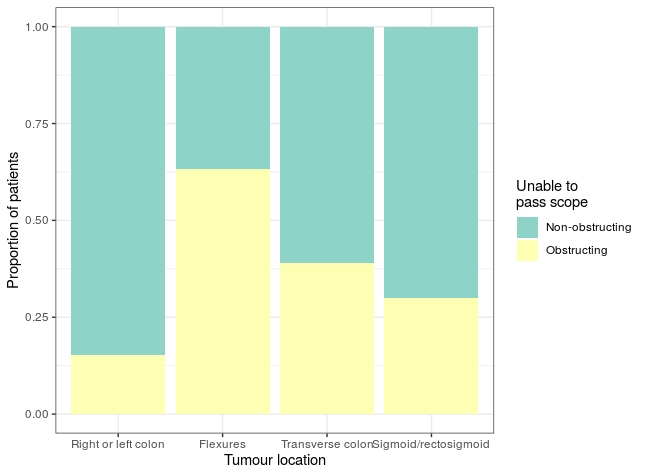
**

**Supplementary figure 2.** Trace plots for the fixed effects parameters in the primary model


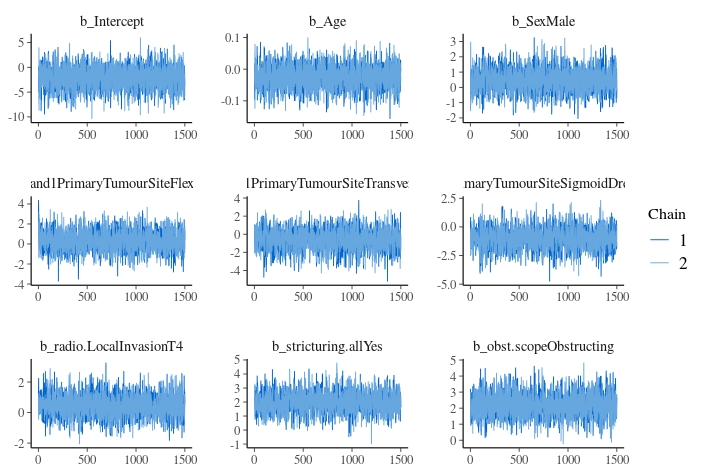


A common check for the Markov Chain Monte Carlo sampler convergence is the trace plot. The plots above show the behaviour of the simulations (i.e., the chains) used to approximate the posterior distribution, where the x-axis represents the number of iterations and the y-axis the value of the parameter. To ensure the reliability of a finite set of samples, we routinely run several chains, i.e. we start the procedure at different random initial starting points and check whether the different chains have converged to stable values. In the figure above, the chains appear to be indistinguishable except for random noise and have converged to stable values. If the chains had not converged, the trajectories of the chains would be in different directions.

**Supplementary figure 3.** Posterior predictive checks for the primary model of factors associated with postoperative pulmonary complications (summarised in Figure 1, and Table 4)


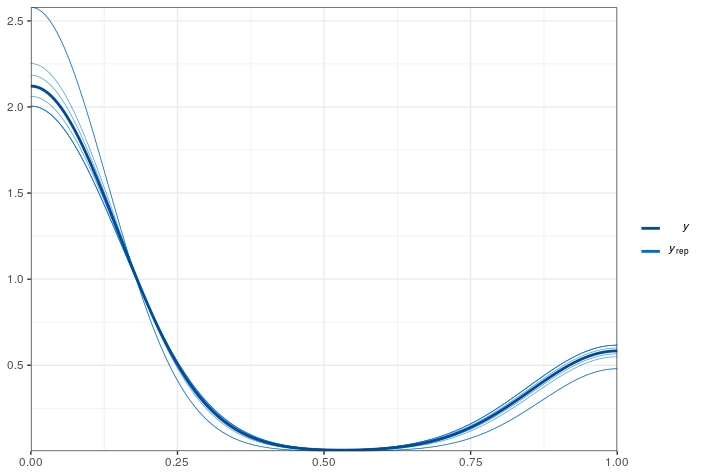


Lines labelled ‘yrep’ refer to the posterior predictive values generated by the model, and the black solid line are the observed data.

**Appendix A.** **Full statistical methodology**

Parametric data were summarised with mean and standard deviation and differences between groups tested using Student’s T-test. Non-parametric data were summarised with medians and interquartile ranges and differences between groups were tested using the Mann-Whitney U test. The χ2 test was used for categorical data.

*Methodology used for Bayesian hierarchical mixed effects modelling*

For all models, non-informative priors were used with sensitivity analyses done using weakly informative priors and with different chain initiation points and Markov-Chain Monte Carlo sampler chain lengths. Models only included factors that occurred before the outcome of interest. Model convergence was assessed using the Rhat statistic. The ratio of between-chain variability and the within-chain variability should be approximately 1 when sampling from the posterior, so an R statistic of approximately 1 for each parameter is an indication that the model has converged. In addition, visual check of the trace plots for the fixed effects parameters gives an indication of the model convergence. Model fit was assessed by how well it matched the observed data using the posterior predictive checks. If the predicted and observed data had similar distributions, it was concluded that the model had a reasonable fit. For data with greater than 5% missingness, we pre-planned to add a separate category level for ‘missing’ data within the mixed-effects model to avoid loss of data; otherwise missing data were excluded from the model.

Appendix B. STROBE Statement—Checklist of items that should be included in reports of ***case-control studies***

|  | Item No | Recommendation | Page No |
| --- | --- | --- | --- |
| **Title and abstract** | 1 | (*a*) Indicate the study’s design with a commonly used term in the title or the abstract | 1 |
|  |  | (*b*) Provide in the abstract an informative and balanced summary of what was done and what was found | 5 |
| Introduction | | | |
| Background/rationale | 2 | Explain the scientific background and rationale for the investigation being reported | 7 |
| Objectives | 3 | State specific objectives, including any prespecified hypotheses | 7 |
| Methods | | | |
| Study design | 4 | Present key elements of study design early in the paper | 8 |
| Setting | 5 | Describe the setting, locations, and relevant dates, including periods of recruitment, exposure, follow-up, and data collection | 8 |
| Participants | 6 | (*a*) Give the eligibility criteria, and the sources and methods of case ascertainment and control selection. Give the rationale for the choice of cases and controls | 8-9 |
|  |  | (*b*) For matched studies, give matching criteria and the number of controls per case | 8 |
| Variables | 7 | Clearly define all outcomes, exposures, predictors, potential confounders, and effect modifiers. Give diagnostic criteria, if applicable | 9-10 |
| Data sources/ measurement | 8* | For each variable of interest, give sources of data and details of methods of assessment (measurement). Describe comparability of assessment methods if there is more than one group | 9-10 |
| Bias | 9 | Describe any efforts to address potential sources of bias | 10-11 |
| Study size | 10 | Explain how the study size was arrived at | 8 |
| Quantitative variables | 11 | Explain how quantitative variables were handled in the analyses. If applicable, describe which groupings were chosen and why | 9-11 |
| Statistical methods | 12 | (*a*) Describe all statistical methods, including those used to control for confounding | 10-11 |
|  |  | (*b*) Describe any methods used to examine subgroups and interactions | 11 |
|  |  | (*c*) Explain how missing data were addressed | 10 |
|  |  | (*d*) If applicable, explain how matching of cases and controls was addressed | 8-9 |
|  |  | (*e*) Describe any sensitivity analyses | 11 |
| Results | | | |
| Participants | 13* | (a) Report numbers of individuals at each stage of study—eg numbers potentially eligible, examined for eligibility, confirmed eligible, included in the study, completing follow-up, and analysed | 12 + Figure 1 |
|  |  | (b) Give reasons for non-participation at each stage |  |
|  |  | (c) Consider use of a flow diagram |  |
| Descriptive data | 14* | (a) Give characteristics of study participants (eg demographic, clinical, social) and information on exposures and potential confounders | 12 + Table 1+3 |
|  |  | (b) Indicate number of participants with missing data for each variable of interest | Table 1+3 |
| Outcome data | 15* | Report numbers in each exposure category, or summary measures of exposure | Table 2 |

| Main results | | 16 | (*a*) Give unadjusted estimates and, if applicable, confounder-adjusted estimates and their precision (eg, 95% confidence interval). Make clear which confounders were adjusted for and why they were included | 14-15, Table 4 |
| --- | --- | --- | --- | --- |
|  |  |  | (*b*) Report category boundaries when continuous variables were categorized | 15, Table 4+6 |
|  |  |  | (*c*) If relevant, consider translating estimates of relative risk into absolute risk for a meaningful time period | N/A |
| Other analyses | 17 | Report other analyses done—eg analyses of subgroups and interactions, and sensitivity analyses | | Supplement |
| Discussion | | | | |
| Key results | 18 | Summarise key results with reference to study objectives | | 15 |
| Limitations | 19 | Discuss limitations of the study, taking into account sources of potential bias or imprecision. Discuss both direction and magnitude of any potential bias | | 18 |
| Interpretation | 20 | Give a cautious overall interpretation of results considering objectives, limitations, multiplicity of analyses, results from similar studies, and other relevant evidence | | 18 |
| Generalisability | 21 | Discuss the generalisability (external validity) of the study results | | 17-18 |
| Other information | | | | |
| Funding | 22 | Give the source of funding and the role of the funders for the present study and, if applicable, for the original study on which the present article is based | | Cover page (1) |

*Give information separately for cases and controls.

**Note:** An Explanation and Elaboration article discusses each checklist item and gives methodological background and published examples of transparent reporting. The STROBE checklist is best used in conjunction with this article (freely available on the Web sites of PLoS Medicine at http://www.plosmedicine.org/, Annals of Internal Medicine at http://www.annals.org/, and Epidemiology at http://www.epidem.com/). Information on the STROBE Initiative is available at http://www.strobe-statement.org.
